# Introduction to the special issue: the two sides of hemp: medical and industrial

**DOI:** 10.1186/s42238-024-00237-9

**Published:** 2024-07-16

**Authors:** Nirit Bernstein

**Affiliations:** Agricultural Research Organization – Volcani Institute, Rishon LeZion, Israel

Hemp, a member of the *Cannabis sativa* L. species, is an annual herbaceous plant belonging to the genus Cannabis of the Cannabinaceae family (Cerino et al. 2021). It contains higher contents of fibers and lower concentrations of the intoxicating THC than ‘drug-type’ cannabis. It is one of the earliest crops cultivated by mankind, and boasts a rich and diverse history spanning thousands of years. Hemp’s native origin appears to be Eurasia, and archaeological and historical evidence suggests that it was cultivated as early as 8000 BCE, with distribution around the world primarily as a fiber crop (Frassinetti et al. 2018). It is a multifunctional crop, with great industrial, nutritional, medical, and environmental potential.

## Industrial applications

Hemp has long been valued for its strong and durable fibers, which have been used historically in textiles, paper, ropes, and construction materials (Rupasinghe et al. 2020). Advances in processing techniques and fiber technology could further enhance the versatility and performance of hemp-based materials, opening up new markets and applications.

## Nutritional and functional foods

Hemp seeds and hemp-derived products are gaining increased popularity as nutritious and functional foods. Hemp seeds are rich in protein, essential fatty acids, vitamins, and minerals (Montero et al. 2023), and as consumer awareness of the health benefits of hemp-based foods grows, so does the potential for the development of hemp-based functional-food dietary supplement products. Hemp oil and hemp seed oil are being explored also in culinary applications for their unique flavor and nutritional profile.

## Medicinal and therapeutic applications

Hemp possesses a range of secondary metabolite phytochemicals, including cannabinoids, terpenes, and flavonoids, which have sparked interest in its potential medical applications (Legare et al. 2022). While hemp was not primarily cultivated for its medicinal properties, certain cannabinoids found in the plant, notably the non-intoxicating cannabidiol (CBD), one of the most abundant cannabinoids in hemp, have garnered attention for their potential therapeutic effects, and are currently the subject of extensive research. As our understanding of the endocannabinoid system and the interactions between cannabinoids and other biological pathways and phytochemicals deepens, so does the potential for the development of new hemp-derived medications and treatments.

## Environmental sustainability

Hemp cultivation offers numerous environmental benefits, including soil remediation and carbon sequestration, making it an attractive option for sustainable agriculture. The high ability of hemp roots to take up heavy metals and other soil contaminants has fostered its use in phytoremediation; its rapid growth makes it a suitable source for production of renewable resources for various industries (Kaur and Kander 2023), contributing to a more self-sustaining economy and mitigating environmental impact.

## Regulatory and legal developments

The regulatory landscape surrounding hemp is evolving rapidly, with changes in laws and regulations opening up new opportunities for research, cultivation, and commercialization. As governments around the world recognize the medical, economic and environmental potential of hemp, there is a trend towards legalization and deregulation of hemp cultivation and processing. Some countries define hemp as a cannabis plant containing less than a specified concentration of THC (e.g., 0.3%, 0.5%) regardless of its botanical classification. Clear and consistent regulations are essential to support the growth of the hemp industry while ensuring consumer safety and compliance with the law.

Overall, the future of the hemp industry looks promising, with opportunities for innovation and sustainable development across a wide range of sectors. As scientific understanding and public acceptance of hemp continue to grow, the potential of hemp becomes increasingly apparent. Realization of this potential will depend on continued research, innovation, and collaboration across different sectors.

This special issue collates manuscripts on the topic of hemp plant science, genetics, chemistry, regulation, and medical and industrial uses. Encompassing contributions from a variety of disciplines, it represents the type of scholarship conducted to-date in this growing field.

*The following section provides a brief outline of the content of each manuscript and its main contribution.*

**Henry et al. (**2020**)** present a targeted genetic assay and algorithms related to sub-genus classification in cannabis including hemp sub-classes. Cannabis accessions were investigated using 23 polymorphic SNP (Single Nucleotide Polymorphism) markers associated with key traits including cannabinoid and terpenoid expression, and fiber and resin production. The results offers insight into cannabis population structure, phylogenetic relationship, population genetics and correlation to secondary metabolite concentrations.

**Berthold et al. (**2020**)** describe an ultra-performance liquid chromatography-tandem mass spectrometry (UPLC-MS/MS) method, with a short run time of 6 min, for the quantification of twelve cannabinoids in hemp samples. This method was applied in a trial regulatory study, and the results revealed that the sampling and analyses methods may influence the detected cannabinoid concentrations, and hence the legality of a hemp crop.

**Huang et al. (**2023**)** characterized the phytochemical profile of industrial hemp roots, and studied the anti-inflammatory activities of hemp roots extract. Six cannabinoids, and 26 non-cannabinoid compounds were identified in the root extracts; and cannabinoids as well as seven non-cannabinoid compounds from root extracts exerted promising anti-inflammatory effects.

**Torres et al. (**2022**)** investigated methodologies for high-throughput sex identification genotyping in cannabis plants, including hemp, using both novel and literature marker sets. They demonstrated a robust high-throughput duplex TaqMan qPCR assay for identification of male-specific genomic signatures, using a novel MADC2 (male-associated DNA from Cannabis) qPCR probe. The viability of using nearby regions to MADC2 with novel primers were demonstrated as alternative assays.

**Filer (**2020) reviewed the role of Minnesota wild hemp in early cannabinoid discovery. Studies conducted in the early 1940’s identified that Minnesota wild hemp served a critical role not only as a crucial fiber plant during World War II but also as a key botanical source for cannabinoid chemistry discoveries.

**Falkner et al. (**2023**)** performed a content analysis to analyze terms and definitions used by tribal and state hemp producers, the USDA hemp producer license, and the 2014 state pilot plans. The results identified discrepancies among different hemp production plans, that were intensified by extending the 2014 Farm Bill language into the 2018 Farm Bill timeframe.

**Burton et al. (**2022**)** review and discusses opportunities for designing a supply chain for industrial hemp, focused on hemp seed and its components for food applications. Market opportunities for industrial hemp products are discussed, as well as growth, harvest, storage conditions, and nutritional properties of hemp seed required to produce value-added food ingredients.

**Johnson et al. (**2023**)** performed a laboratory and market analysis to evaluate the adherence of hemp products to legal regulations in the US. The results revealed that some hemp products are inaccurately labeled andcontain higher levels of THC than would be allowed legally for recreational (“adult”) use. This raises consumer safety concerns involving consuming intoxicating products.

**Johnson et al. (**2022**)** analyzed the CBD content in commercial hemp-derived CBD products available in Central Kentucky from both online and local retailers. The results demonstrated that CBD content in over-the-counter CBD products is often inconsistent with the label claims.

**Vasudevan and Stahl (**2020) evaluated the efficiency of cannabinoids (CBG/CBD) infused mouthwash products against dental plaque bacteria. The cannabinoids infused products showed a similar bactericidal efficacy as that of chlorhexidine 0.2%, and no significant difference was observed between CBD- and CBG-mouthwash. These in vitro results demonstrate the potential of cannabinoids for developing efficient and safer mouthwash products that do not contain fluoride and alcohol.

**Zadik-Weiss et al. (**2020**)** conducted a literature review to evaluate the potential of domestic cats as a spontaneous model for Alzheimer’s disease. In line with the One-Health concept, feline cognitive dysfunction was identified as a promising model for human Alzheimer’s disease. The authors suggest that performing a clinical trial in aging domestic cats, for researching the benefits of CBD (often derived from hemp) for both conditions, can promote the treatment of these two conditions in both humans and pets.

**Mazza (**2021) assessed the efficacy and adverse events of short- and long-term medical cannabis treatment for fibromyalgia syndrome, in patients resistant to conventional therapy, which were treated with medical cannabis of various THC and CBD contents. The results suggest medical cannabis as an alternative treatment for patients unresponsive to conventional therapy. However, its application may be limited by the incidence of nonserious adverse events.

**Trac et al. (**2021**)** explored the literature related to the potential anticancer therapeutic activity of the cannabinoid-quinone HU-331, in purified systems, cancer cell lines, and animal models. The available studies indicates that HU-331 has promising anticancer properties, and support its further evaluation for the treatment of cancer and possibly other diseases.

**Bilge and Ekici (**2021) report on a 2-year study with CBD-enriched cannabis for the treatment of autism, and review the latest studies regarding CBD treatment in autism spectrum disorder. The most substantial improvements were found in behavioral problems, described in 20–70% of the patients under the low doses of CBD-enriched cannabis used (an average daily dosage of 0.7 mg/kg/day CBD, with no significant side effects.

**Hall et al. (**2023**)** were first to assess the tolerability of CBD in former elite athletes, a population that is susceptible to chronic pain duo to disabling injuries, and also highly trained to assess medication tolerability concerns. Topical CBD administration was well tolerated by this population and resulted in only minor adverse effects, and there was a significant improvement in pain levels and pain-related disability.

**Wurz et al. (**2022**)** evaluated cannabinoid kinetics and impairment patterns associated with the use of Δ^8^-THC. Cannabinoid kinetic patterns after Δ^8^-THC vaporizing were similar to those observed for ∆^9^-THC; and hemp-derived Δ^8^-THC and Δ^9^-THC displayed similar impairment profiles, suggesting that use of Δ^8^-THC products may carry the same risks as Δ^9^-THC products.

**Wyse and Luria (**2021) reviewed intellectual property rights (IPR) data for medical cannabis, including hemp, with the aim of advancing applied cannabis research by providing insights into medical cannabis patenting. The results present characteristics of the intellectual property landscape of the medical cannabis field, with the IPR data shown to be distributed along the entire medical cannabis supply chain, following the upstream–midstream–downstream paradigm.

**Stats et al. (**2023**)** evaluated the causes for the collapse of the CBD-hemp cultivation industry in Yuma County, Arizona, USA. Major factors were identified for this trend: lack of knowledge on the life cycle of the hemp plant, noncompliance with THC limits, poor seed sources and inconsistent genetics, and susceptibility to diseases such as beet curly top virus, crown root rot, and *Pythium*.

**Sommano et al. (**2022**)** evaluated the recent guidelines for receiving a cannabis production license in Thailand, including hemp, and demonstrated that the announced law is in-line with regulations in many countries in terms of prevention of misuse and security. The authors recommend that the regulation in Thailand should be revisited to promote cannabis as an economic crop, and for tighter control and prevention of misuse.

**Quansah Amissah (**2022**)** examined local cannabis production, industrial hemp facilities, and the potential benefits of industrial and medical hemp in Ghana. Based on the availability of resources, and the potential benefits of hemp-based drugs to Ghanaians, the most promising and feasible option for the government to invest in was identified to be exploitation of medicinal hemp.

**Quansah Amissah (**2023**)** focuses on Ghana’s potential to establish a hemp industry, based on its suitable climate, available agricultural resources, and the policies that support enhancement of the agricultural sector. They concluded that development of a Hemp industry could drive economic growth, create job opportunities and promote sustainable development in Ghana.

## Conclusion

With its environmentally friendly characteristics, medicinal and nutritional benefits, and potential economic value, hemp stands as a promising resource for numerous industries, and a subject of increasing interest in modern agriculture and sustainability initiatives.

## References

[CR1] Berthold EC, Yang R, Sharma A, Kamble SH, Kanumuri SR, King TI, Popa R, Freeman JH, Brym ZT, Avery BA. McCurdy,.CR. (2020). Regulatory sampling of industrial hemp plant samples (*Cannabis sativa* L.) using UPLC-MS/MS method for detection and quantification of twelve cannabinoids. *J. Cannabis Res* 2, 42. 10.1186/s42238-020-00050-0. 10.1186/s42238-020-00050-0 PMC7819288 33526142

[CR2] Bilge S, Ekici B (2021). CBD-enriched cannabis for autism spectrum disorder: an experience of a single center in Turkey and reviews of the literature. J Cannabis Res.

[CR3] Burton RA, Andres M, Cole M, Cowley JM, Augustin MA. Industrial Hemp seed: from the field to value-added food ingredients. J Cannabis Res. 2022;4. 10.1186/s42238-022-00156-7. 10.1186/s42238-022-00156-7 PMC9338676 35906681

[CR4] Cerino P, Buonerba C, Cannazza G, D’Auria J, Ottoni E, Fulgione A, Di Stasio A, Pierri B, Gallo A (2021). A review of Hemp as food and nutritional supplement. Cannabis Cannabinoid Res.

[CR5] Falkner A, Kolodinsky J, Mark T, Snell W, Hill R, Luke A, Shepherd J, Lacasse H (2023). The reintroduction of hemp in the USA: a content analysis of state and tribal hemp production plans. J Cannabis Res.

[CR6] Filer CN (2020). Minnesota wild hemp: a crucial botanical source in early cannabinoid discovery. J Cannabis Res.

[CR7] Frassinetti S, Moccia E, Caltavuturo L, Gabriele M, Longo V, Bellani L, Giorgi G, Giorgetti L. (2018). Nutraceutical potential of hemp (*Cannabis sativa* L.) seeds and sprouts. *Food Chem* 2018;262:56–66. 10.1016/j.foodchem.2018.04.078. 10.1016/j.foodchem.2018.04.078 29751921

[CR8] Hall N, James B, Bhuiyan MAN, Crane E, Falgout C, Murnane KS (2023). Topical cannabidiol is well tolerated in individuals with a history of elite physical performance and chronic lower extremity pain. J Cannabis Res.

[CR9] Henry P, Khatodia S, Kapoor K (2020). A single nucleotide polymorphism assay sheds light on the extent and distribution of genetic diversity, population structure and functional basis of key traits in cultivated north American cannabis. J Cannabis Res.

[CR10] Huang S, Li H, Xu J, Zhou H, Seeram NP, Ma H, Gu Q (2023). Chemical constituents of industrial hemp roots and their anti-inflammatory activities. J Cannabis Res.

[CR12] Johnson E, Kilgore M, Babalonis S (2022). Label accuracy of unregulated cannabidiol (CBD) products: measured concentration vs. label claim. J Cannabis Res.

[CR11] Johnson L, Malone M, Paulson E, Swider J, Marelius D, Anderson S, Black D (2023). Potency and safety analysis of hemp delta-9 products: the hemp vs. cannabis demarcation problem. J Cannabis Res.

[CR13] Kaur G, Kander R (2023). The sustainability of Industrial Hemp: a literature review of its economic, environmental, and social sustainability. Sustainability.

[CR14] Legare CA, Raup-Konsavage WM, Vrana KE (2022). Therapeutic potential of cannabis, cannabidiol, and cannabinoid-based pharmaceuticals. Pharmacology.

[CR15] Mazza M (2021). Medical cannabis for the treatment of fibromyalgia syndrome: a retrospective, open-label case series. J Cannabis Res.

[CR16] Montero L, Ballesteros-Viva sD, Gonzalez-Barrios AF, Sánchez-Camargo ADP (2023). Hemp seeds: nutritional value, associated bioactivities and the potential food applications in the Colombian context. Front Nutr.

[CR17] Quansah Amissah R (2022). Ghana’s preparedness to exploit the medicinal value of industrial hemp. J Cannabis Res.

[CR18] Quansah Amissah R (2023). The potential for Ghana to become a leader in the African hemp industry. J Cannabis Res.

[CR19] Rupasinghe HPV, Davis A, Kumar SK, Murray B, Zheljazkov VD (2020). Industrial Hemp (*Cannabis sativa* subsp. *sativa*) as an emerging source for Value-Added Functional Food Ingredients and nutraceuticals. Molecules.

[CR20] Sommano SR, Tangpao T, Pankasemsuk T, Ponpanumas V, Phimolsiripol Y, Rachtanapun P, Prasad SK. Growing ganja permission: a real gate-way for Thailand’s promising industrial crop? J Cannabis Res. 2022;6(4):1–10. 10.1186/s42238-022-00121-4. 10.1186/s42238-022-00121-4 PMC8898406 35249552

[CR21] Stats AK, Sweat KG, Masson RN, Conrow CD, Frazier AE, Leung MCK (2023). The Desert Whale: the boom and bust of hemp in Arizona. J Cannabis Res.

[CR23] Torres A, Pauli C, Givens R, Argyris J, Allen K, Monfort A, Gaudino RJ (2022). High-throughput methods to identify male Cannabis sativa using various genotyping methods. J Cannabis Res.

[CR22] Trac J, Keck JM, Deweese JE (2021). Cannabidiol oxidation product HU-331 is a potential anticancer cannabinoid-quinone: a narrative review. J Cannabis Res.

[CR24] Vasudevan K, Stahl V (2020). Cannabinoids infused mouthwash products are as effective as chlorhexidine on inhibition of total-culturable bacterial content in dental plaque samples. J Cannabis Res.

[CR25] Wurz GT, Montoya E, DeGregorio MW (2022). Examining impairment and kinetic patterns associated with recent use of hemp-derived ∆^8^-tetrahydrocannabinol: case studies. J Cannabis Res.

[CR26] Wyse J, Luria G (2021). Trends in intellectual property rights protection for medical cannabis and related products. J Cannabis Res.

[CR27] Zadik-Weiss L, Ritter S, Hermush V, Asher N, Avital A, Or R (2020). Feline cognitive dysfunction as a model for Alzheimer’s disease in the research of CBD as a potential treatment—a narrative review. J Cannabis Res.

